# Context-Specific Habituation: A Review

**DOI:** 10.3390/ani11061767

**Published:** 2021-06-12

**Authors:** Andrea Dissegna, Massimo Turatto, Cinzia Chiandetti

**Affiliations:** 1 Department of Life Sciences, University of Trieste, 34136 Trieste, Italy; andrea.dissegna@phd.units.it; 2 Center for Mind/Brain Sciences (CIMeC), University of Trento, 38068 Rovereto, Italy; massimo.turatto@unitn.it

**Keywords:** associative learning, plasticity, evolution, habituation model

## Abstract

**Simple Summary:**

Habituation reflects the ability to learn to ignore irrelevant stimuli, which form the vast majority of the sensory input impinging on any organism’s sensory systems at a given moment. However, although habituation is often described as one of the simplest forms of learning affected only by the stimulus features, such as frequency or intensity, in fact evidence exists showing that habituation can be specific for the context in which it takes place. This shows that habituation, in some cases, exhibits an associative nature, and that the underlying learning mechanism is more sophisticated than previously believed.

**Abstract:**

Habituation consists of the progressive response decrement to a repeated stimulation, a response decline that is not accounted for by sensory or motor fatigue. Together with sensitization, habituation has been traditionally considered to be a prototypical example of non-associative learning, being affected only by the features of the stimulation, as for instance its intensity or frequency. However, despite this widespread belief, evidence exists showing that habituation can be specific to the context of the stimulation, thus suggesting that habituation can have an associative nature. Such an unexpected characteristic of habituation was in fact predicted by a theoretical model of associative learning proposed by Wagner in a series of works that appeared in the late 1970s. Here, we critically review the experimental data that since then have been accumulated in support of this hypothesis. What emerges from the literature is that context-specific habituation is common to several animal species and that the ability to form an association between the habituating stimulus and its context is independent of the complexity of the animal’s nervous system. Finally, context-specific habituation is observed for a variety of organism’s responses, ranging from visceral to motor and mental activities.

## 1. Introduction

It is late at night and you feel sleepy. After putting down the book you were reading you turn the light off, and in a few moments your consciousness fades away. You have fallen asleep despite the distinct ticking of your bedside clock, which you did not notice at all. How is that possible? The answer is habituation, a widespread phenomenon showing that animals usually cease to respond to repetitive stimuli, especially if irrelevant, and that the nervous system is capable of learning to filter out certain sensory inputs [1]. The next day you have guests, and you kindly offer them your bedroom for the night. You then go sleeping on the sofa, and take your bedside clock with you. It is bedtime, your guests have retired and you feel like sleeping, but this time you cannot avoid noticing the ticking of the clock, which keeps you awake the whole night. What has happened? Isn’t this the same clock you were able to ignore in your bedroom? Apparently yes, but sometimes the nervous system reacts in a different way to familiar stimuli when encountered in a different place. To put it differently, habituation can be specific for the context in which it has occurred. Thus, although habituation is usually considered a prototypical example of non-associative learning, in which the vanishing of the response is determined only by the characteristics of the stimulation, in fact habituation can be context specific, revealing that it can arise from an associative learning process that takes into account also the surrounding environment.

**Figure 1:**
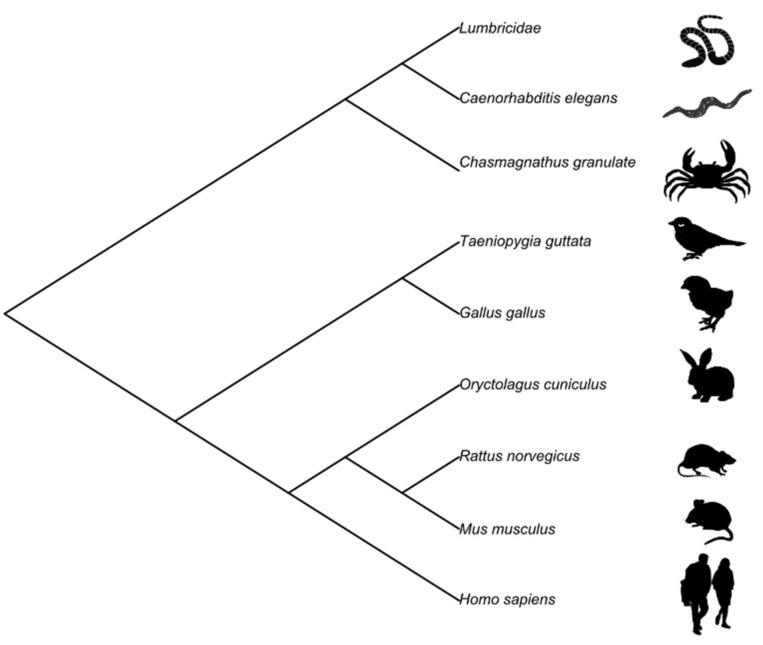
Cladogram of the species showing context-specific habituation.

In agreement with this notion, we will review existing evidence of context-specific habituation, showing that this phenomenon is common to different animal species (see [2]). Furthermore, and quite surprisingly, this sophisticated learning ability is not dependent on the complexity of the animal’s neural system, as context-specific habituation has been shown both in human beings, who possess a brain composed of about 80 billion neurons, and in different types of worms, whose behavior is controlled by a few hundred neurons (see Figure 1).

However, before reviewing the experimental studies providing support to the associative nature of habituation, it may be useful to elucidate on which theoretical grounds habituation should be, under certain circumstances, context specific.

## 2. A Context-Specific Theory of Habituation: Wagner’s Model

Historically, three main different models of habituation have been proposed: the stimulus-model comparator theory [3], the dual-process theory [4], and the gnostic-unit theory [5]. The models show both commonalities and differences [1], but only Wagner’s model postulates that habituation can be context specific, and therefore it will be presented more in detail. Still, it may be worth also mentioning the key notions of both the stimulus-model comparator theory devised by Sokolov [3,6], and the dual-process theory elaborated by Groves and Thompson [7]. As for the former, it was mainly devised to account for habituation of the orienting response (OR), and its key mechanism consists of a comparison process between the current sensory input and a neural model of past stimulation. When the result of the comparison is a mismatch, an OR toward the new stimulation is triggered; by contrast, the more the stimulus matches the neural model, the more the OR is inhibited, and habituation arises [3,6]. The dual-process theory, instead, was developed on the basis of the acute-spinal cat model and grounded on the results of physiological recordings showing the existence of two main types of neurons in the spinal cord, those that reduce their response with stimulus repetition, causing habituation, and those that before habituating show an initial response increment called sensitization. The two classes of neurons form two independent systems, the S-R pathway leading to habituation, and the state-system controlling the arousal level and producing sensitization. Activity from both systems is then integrated at some synaptic level to determine the final response to the stimulation, which in general decreases with repetition [7]. As anticipated, neither of these two theories anticipate that habituation could be context specific.

Let us now turn to Wagner’s model, which was originally elaborated and presented in three consecutive articles that appeared in 1976, 1978, and 1979, respectively [5,8,9], in the context of a more general conceptualization of how stimuli are differently processed in memory. The model was particularly inspired by the previous work of Konorski [10], who also proposed a similar habituation mechanism, and of Aktinson and Shiffrin [11] on the structure of memory, in which the functional distinction between short-term memory (STM) and long-term memory (LTM) was delineated. Although further developments of the original model have been proposed by Wanger and his collaborators [12,13], the basic principles governing habituation, schematically outlined below, remained those enounced in his seminal works, where more detailed explanations of the underlying mechanisms can be found [5,8,9]. 

Pivotal to Wagner’s model is the idea that LTM can be conceptualized as a set of interconnected nodes forming an associative network, with each node (also known as a gnostic unit) coding for a given knowledge or stimulus representation. In the LTM the vast majority of gnostic units are inactive, whereas the STM corresponds to the subset of these units that are temporarily active [10,11]. The model also postulates that when a stimulus is initially presented, the corresponding representation becomes fully active in the STM, which strongly also activates the response generator. However, because of the transient nature of the STM, the activation of the stimulus representation will decay over time. A key notion of the model is that while the representation is active it prevents a subsequent instance of the same stimulus to activate its representation at full strength again, and consequently the response elicited by the repeated stimulus becomes weaker. In other words, “…the likelihood of a response on trial *n* of a habituation sequence appears to be depressed by a short-term refractory-like effect generated by recent trial events…” ([8], p. 109), an effect that Wagner termed “priming” of the STM. This simple refractory-like mechanism is capable of explaining one of the main features of habituation, namely that it is directly proportional to the frequency of stimulation. Indeed, each occurrence of the stimulus will elicit a self-generated priming, which however decays over time. Hence, short-term habituation, namely habituation observed within a sequence of stimulation, is usually stronger the shorter the inter-stimulus-interval (ISI) or, conversely, the higher the frequency of stimulation.

It is worth noting, however, that the ISI has another important consequence on habituation. Indeed, the longer the ISI the more the stimulus representation remains active in the STM without being disrupted or weakened by subsequent events (priming of the STM). This, in turn, also increases the probability that such representation will enter in associations with other contextual stimuli, and that these associations will be transferred and consolidated in the LTM [14,15]. Hence, long-term habituation, measured across different sequences of stimulation, should be stronger the longer the ISI [14,15]. Crucially, the contextual cues encountered again following the initial habituation phase can retrieve or prime representation of the associated habituating stimulus in the STM (associatively-generated priming). As a consequence, if the stimulus re-occurs in the same context the corresponding response retains a certain degree of habituation. Yet, if the stimulus appears in a different context, with which no previous associations were formed, contextual cues will not prime the stimulus representation in the STM, and the previously habituated response will recover. Hence, because of associative mechanisms analogous to those involved in Pavlovian conditioning, the Wagner model predicts that long-term habituation is context specific.

Although the model devised by Wanger is the only one that explicitly posits that long-term habituation is controlled by stimulus-context associations, in fact the model proposed by Sokolov [3,6] could also be compatible with context-specific habituation. To the extent that the neural model of the stimulus is defined not only by the specific stimulus features, as originally proposed, but incorporates also some characteristics of the surrounding environment, the stimulus appearance in a new context will cause a mismatch between the sensory input and the stored neural model of past stimulation, and consequently a recovery of the previously habituated response. On the contrary, contextual habituation is not easily accommodated in Groves and Thompson’s dual-process theory [7], which does not postulate the existence of associative links between the stimulus and its context.

In fact, a fourth alternative account of habituation has been recently proposed by Hall and Rodriguez [16,17], which postulates that habituation for a given stimulus does not arise the more the stimulus is predicted (or primed), as assumed for example by Sokolov’s or Wagner’s models; rather, habituation is the consequence of a progressive reduction in the stimulus salience (which determines the ability to evoke its unconditioned response) due to the fact that across repetitions the stimulus is followed by no consequences. In other words, this account emphasizes the fact that habituation would depend on the predictiveness of the habituating stimulus rather than on its predictability. The model does not exclude the possibility that the context can modulate habituation but interprets this effect as an instance of occasion setting rather than evidence that associations have been formed between the context and the habituating stimulus. 

## 3. Methods to Investigate Context-Specific Habituation

Three different approaches have been traditionally used to show that habituation can be specific for the context of stimulation. The most straightforward is the context-change method, whereby the context changes from the training to the test phase. Evidence of context-specific habituation is found when the response habituated during training recovers in a different context during the test. A second approach to reveal the associative nature of habituation relies on the extinction phenomenon. Here the logic is that during training the stimulus automatically forms associations, stored in the LTM, with the surrounding environmental cues [8]. When the context is further encountered in the future, these associations prime the stimulus (or anticipate its arrival) in the STM, thus maintaining previous habituation. However, if after habituation the organism is repeatedly exposed to the same context without the stimulus (the extinction condition), the previously formed associations will extinguish, and the habituated response will recover when the stimulus is reintroduced in the same context. By contrast, if the stimulus is omitted altogether with the context (the control condition), the stimulus-context associations will be retained in the LTM, so that habituation will remain effective when the stimulus occurs again in the same context. A third method is based on the latent inhibition phenomenon reported in conditioning studies [18]. In this phenomenon, the association between the conditioned and the unconditioned stimuli (CS and US) is delayed when the CS is pre-exposed in isolation before being paired with the US. One of the viable explanations is that, during the pre-exposure phase, the CS becomes associated with contextual cues, which then reduce its ability to enter in association with the US. Alternatively, during the pre-exposure phase the animal learns that the context predicts no consequences, namely that no US would follow. In the same vein, if habituation is context specific, then by presenting the context alone in a pre-exposure phase it will be less associated with the to-be habituated stimulus during training. Hence, at test the context would not activate, or would activate to a lesser extent, the stimulus representation in the STM, and the retention of habituation will be compromised as compared to when the pre-exposed context is different from that used during training.

To conclude this brief overview of the rationale behind the main methods used to address the associability of habituation, it should be worth mentioning that at least two alternative phenomena can explain the disruption of habituation when the context changes. One is that the presentation of a stimulus in a different environment may simply alter the stimulus representation. This would make the stimulus essentially new to an organism, leading to a disruption of the ongoing habituation. A second one is that a sudden change of the context of stimulation might lead to a neophobia reaction. This in turn increases the animal’s arousal and propensity to respond to any stimulus, a result that could mimic a context-specificity effect [19,20,21]. To rule out this possible confound, in many studies animals have been given the possibility to familiarize with the new context before the test (see Table 1).

## 4. Context-Specific Habituation in Humans

Evidence of context-specific habituation in humans (*Homo sapiens*) is rather scant. In one of the few studies that have addressed this issue, Turatto, Bonetti, and Pascucci [30] investigated whether habituation of attentional capture, a covert component of the OR, is context specific or generalizes across different contexts. In three consecutive days, participants performed a visual discrimination task in focused attention, while a visual onset distractor appeared in the display, which also defined the context of stimulation. The results showed that on day 1 the capture of attention triggered by a repetitive visual onset distractor was subject to habituation. Then, on day 2, participants were divided into two groups: the extinction group performed the same visual discrimination task of day 1, but without the distractor, whereas the control group suspended the task. On day 3, both groups resumed the visual task with the distractor. While habituation of capture was still present in the control group, the attentional capture response recovered in the extinction group, a result consistent with a context-specific habituation view [5,8,9]. Evidence in favor of a context-specific habituation of attentional capture emerged also in a subsequent study [31]. Here the stimuli used to measure habituation of capture were presented over a background consisting, for example, of a countryside landscape. The next day, one group of participants continued the same task in the same context, whereas for another group the context changed, with the background image now depicting an industrial landscape. Habituation of capture was retained in the same-context group but was disrupted in the different-context group. 

Studies investigating the context-specific habituation of different electrophysiological responses have provided less consistent results. An electrodermal study by Churchill, Remington, and Siddle [32] recorded the skin conductance response of participants repeatedly exposed to a geometrical shape projected on a monitor. The authors found the same level of long-term habituation when either local contextual cues—e.g., the background image of the monitor—or global contextual cues—e.g., the furniture in the experimental room—changed between the training and test sessions, thus showing generalization of habituation across different contexts. Similarly, an extinction session with the trained context did not produce a recovery of the habituated response. Schaafsma, Packer, and Siddle [33] studied the role of context in long-term habituation of the skin conductance response to stimuli with different motivational value. Specifically, all participants were exposed to tones and vibrations as habituation stimuli. The authors manipulated the motivational value of either tones or vibrations by instructing participants to press a microswitch at the offset of one of the two types of stimuli. The hypothesis was that instructing participants to perform a specific action in response to one of the two habituation stimuli would increase the amount of processing that one stimulus received from the STM relative to the other. Since stimuli that receive more processing in the STM are more likely to consolidate their association with contextual cues [5,8,9], the authors expected that the context change would produce less long-term habituation retention for the motivationally significant stimulus than for the other one. However, the authors found no effect of context for either of the stimuli.

## 5. Context-Specific Habituation in Non-Human Mammals

An initial evidence of context-specific habituation was reported by Wagner [8] in an unpublished work on rabbits. The author reported lower retention of long-term habituation of the vasoconstriction response to a repeated tone in rabbits that remained in the experimental apparatus between the training and test session compared to animals that returned to their home cage, a pattern of results indicating a disruption of habituation due to the extinction of the context-stimulus association. Several subsequent studies have used the rat as an animal model to study context-specific habituation. Of remarkable importance is the discovery made by Siegel [22] that the associative link between context and habituation is implicated in the regulation of drug tolerance. Tolerance is an instance of habituation because some of the drug effects decrease with its administration. For example, in rats the analgesic effects of morphine declines after repeated injections, leading rats to increase the amount of narcotic to resist pain. Siegel [22] demonstrated that rats injected with morphine in a given context developed a context-specific tolerance for that drug, but tolerance dropped significantly when rats were given the shot in a new context. He also demonstrated context-specific morphine tolerance using latent inhibition and extinction, thus attesting that the associative nature of habituation can interact with the biological processes underlying addiction.

The scenario, however, is a bit more intricate, as some responses appear to be more prone to show context-specific habituation than others. In particular, habituation has been shown to depend on context in the case of inhibitory responses (e.g., lick suppression or bar-press suppression [34,37]; see [20,38] for a null result), whereas there is no evidence for the context-specific habituation of the startle response [34,35] (see also [36] for a study on mice), and conflicting results for different aspects of the OR (null results have been reported in [19]; a positive result in [34]). Specifically, the study of Jordan, Strasser, and McHale [34] has found, in the same animal, evidence of the context-specific long-term habituation of lick and bar-press suppression, but not of the acoustic startle response. Moreover, they found that extinction of context disrupted the long-term habituation of lick-suppression and of the OR to a light. These results suggest that different responses supported by independent neural circuits can be differentially sensitive to the context, in agreement with the hypothesis that habituation does not represent a unitary phenomenon affecting all behaviors in the same fashion [41,42]. 

However, as briefly mentioned above, it is important to stress the fact that not all the response increments observed after a context change are instances of context-specific habituation. For example, Hall and Channell [19] showed that once rats stopped to orient toward a repetitive flashing light, orientation increases again when rats were moved into a new context—apparently supporting the context-specific hypothesis—but this effect disappeared if rats could familiarize with the new context before the test. Hall and Channell [19] hypothesized that when rats were tested in the new context, the OR increased as a consequence of the neophobia induced by the context change [43]. However, contrary to context-specific habituation, response increments due to neophobia or arousal should affect the overall responsivity of the animal [4]. Indeed, a similar response sensitization was measured in a new context also in rats tested for habituation of lick-suppression [20] and neophobia reaction induced by a new flavor [21].

A less tested hypothesis is that context-specific habituation might be modulated by the biological significance of the stimulation. Indeed, stimuli that are more relevant to an organism are more likely to form associative links with the surrounding environment [5,8,9]. In line with this hypothesis, Evans and Hammond [37] showed that long-term habituation of lick suppression elicited by the distress squeal of another rat was context-specific, whereas long-term habituation of the same response caused by an artificial sound with similar acoustical features was not.

## 6. Context-Specific Habituation in Birds

Studies with two types of avian species, zebra finches and chicks of domestic fowl, have provided converging evidence of context-specific habituation in birds. Kruse, Stripling, and Clayton [29] reported for the first time that habituation of a genetic response is context specific. They measured the expression of the *zenk* gene—a specific immediate early gene (IEG) synthetized in the auditory brain areas of zebra finches (*Taeniopygia guttata*)—in birds repeatedly exposed to a conspecific song. The results showed that the expression of this gene decreased when the same song was repeated in the same context. However, when finches listened the familiar song under different light conditions, the synthesis of the *zenk* gene spiked again, suggesting that habituation of its expression was specific for the context in which the song was experienced.

The research with domestic chicks has focused on the development of the associative mechanism underlying context-specific habituation. Chicks of domestic fowl (*Gallus gallus*) are precocial birds that develop almost completely in the egg. Thus, after hatching, they already have enough cognitive and motor skills to be independent of parental care. Chiandetti and Turatto [39] demonstrated that the associative learning process underlying context-specific habituation is also part of this early cognitive equipment. They measured the stop of the wheel-running behavior elicited by a loud sound in 4-day old chicks in four consecutive sessions of stimulation within the same context, comparing this performance with that of groups of chicks for which different aspects of the context were changed after the first two sessions. The degree of generalization vs. specificity of the habituated freezing response to the sound varied with the number of features that the training contexts shared with the test context. In addition, it should be also noted that the increased response observed when the context changed cannot be accounted for by a general increased arousal, as the response measured was the stop of an already ongoing activity (i.e., wheel-running behavior).

Furthermore, a recent study by Turatto, Dissegna, and Chiandetti [40] suggests that the ability to take into account the context of stimulation to filter unwanted sensory input is an innate cognitive ability in chicks. The authors exposed one group of animals in the last stage of their embryonic maturation to a repetitive sound, and then tested their freezing response to an analogous series of sounds in a running wheel two days after hatching. They compared habituation to the sounds in this group of animals with that of another group of chicks exposed to the sounds one day after birth in the running wheel or in a different context, namely in the chicks’ home cage. The results showed that the prenatal group of chicks had a similar disruption of long-term habituation as the chicks trained in the home cage. Nevertheless, their degree of habituation to the sounds was higher than that of an untrained group, attesting that the prenatal stimulation had successfully induced habituation, and that the level of habituation was comparable to that of the group stimulated and tested in two different contexts.

## 7. Context-Specific Habituation in Invertebrates

Despite the relatively simple organization and modest dimension of invertebrate’s nervous systems, the study of habituation in these species has confirmed that this form of learning can be context specific. Here we will briefly review the main studies conducted with the crab (*Chasmagnathus granulate*), the nematode (*Caenorhabditis elegans*) and, more recently, the earthworm of the *Lumbricidae* family [23].

There exists a long tradition of studies concerning the remarkable ability of crabs to form associations between stimuli and context and to flexibly adapt their escape responses. Tomsic and colleagues [24] demonstrated that changing the contextual cues between the training and test phase produced a recovery of habituation of the escape response elicited by a paddle moving above the animal. The authors found the same result when crabs were exposed to the context prior to or following habituation training, thus attesting that the escape response was also sensitive to latent inhibition and extinction [25]. Furthermore, the neurobiological mechanisms regulating context-specific habituation were also investigated by injecting crabs with an inhibitor of protein synthesis (*Cycloheximide)*. Interestingly, injection of the drug before the training blocked the context-specificity of habituation in crabs that could fully retain long-term habituation even when the context changed. By contrast, the injection of the inhibitor after the training impaired the formation of long-term habituation in crabs tested either in the same or different contexts [44,45]. This pattern of results suggests the presence of two distinct cellular processes that lead to context-specific habituation: one responsible for the formation of contextual memories, that was immediately activated as crabs were placed in the training context; the second deputed to the consolidation of long-term habituation triggered by repeated exposure to the stimulus.

The research on crabs has also revealed that the frequency of stimulation is also critical for the emergence of context-specific habituation. For example, it has been suggested that the longer a stimulus representation remains active in the STM before being displaced by the next stimulus occurrence, the more likely it will consolidate its association with the representations of contextual cues in the LTM [15]. In line with this, Hermitte et al. [26] demonstrated that context change produced a recovery of the habituated response in a group of crabs trained with a 171 s inter-stimulus-interval (ISI), but not in a group of crabs trained with a 0 s ISI. Moreover, the injection of the protein inhibitor *Cycloheximide* impaired the retention of long-term memory only in crabs trained at a 171 s ISI, attesting that training with long and short ISIs recruited separate cellular processes (for a replication of this result, see [46]).

However, it remains unclear whether only training with long ISIs results in context-specific habituation. Of considerable importance to this topic is a study in which Rankin [27] investigated context-specific habituation of the tap-withdrawal response in the *C. Elegans*, with stimulations delivered with either a 10 or a 60 s ISI. The context was defined by the presence of a given chemical substance (sodium acetate, NaCh_3_COO) in the petri-dish housing the nematode. The results showed greater retention of habituation at both 10 and 60 s ISI in animals trained and tested in the presence of NaCh_3_COO, as compared with a group trained in the same context but tested in a different one (i.e., plain agar). Rankin also found that context-specific habituation was abolished by latent inhibition and extinction if the animals were exposed to the chemosensory context in the absence of the tap. The fact that context-specific habituation emerged also with the shorter ISI is surprising because with a 10 s ISI long-term habituation is usually not observed in *C. Elegans* [47]. This paradoxical result was found also by Lau, Timbers, Mahmoud, and Rankin [28] in which another chemical substance (diacetyl, C_4_H_6_O_2_) was used as the context. Lau et al. also compared several mutant strains of *C. Elegans* to identify genes involved in context-specific habituation. They found that worms with a mutation in the *nmr-1* gene (NMDA-type glutamate receptor subunit) showed comparable long-term habituation to the non-mutant group when trained and tested in a plain petri-dish, but they showed a lack of context-specific effect in the presence of the olfactory cue, indicating a deficit in their capacity to associate the tap with the context. This suggests that context-specific habituation and long-term habituation involve different biological mechanisms, that long-term habituation does not necessarily require context-stimulus associations, and that the mechanisms underlying context-specific habituation are activated by both short and long ISIs, whereas those responsible for long-term habituation are activated only by long ISIs.

## 8. Conclusions

Textbooks often describe habituation as a prototypical example of non-associative learning consisting of the simple vanishing of basic reflexes for repetitive stimulation (e.g., [48]). Despite this simplistic description, habituation is observed for a multitude of pivotal and complex animal behaviors, such as sexual partner choice [49] and food intake [50]. Furthermore, the underlying cognitive mechanisms of habituation have been the focus of a great bulk of research for almost a century [1]. However, the predominant belief that habituation is an instance of non-associative learning [1,51] may have considerably limited the research efforts devoted to study whether habituation may, instead, have an associative nature. Here we have briefly summarized evidence, from phylogenetically distant species, showing that habituation can be context specific for a large set of responses (see Table 1), and irrespective of the complexity of the nervous systems considered (see Figure 1). Therefore, the ability to take into account the context of stimulation to regulate the organism response must have had a high adaptive value, such that natural selection has favored the spreading of this sophisticated form of learning across different animal and plant species.

Finally, the reviewed results also suggest that context specificity should be included among the features of habituation [1], and that, in our view, the model proposed by Wagner [5,8,9] is perhaps the most exhaustive in accounting for habituation (at least in non-human animals), including its feature of being, for certain types of responses, context specific.

## Figures and Tables

**Table 1:** Evidence of context specificity as a function of behavioral response in different species.

Behavioral Response	Species	Method	Evidence of Context Specificity	Study
Drug tolerance	*Rattus norvegicus*	CC	✓	[22]
Escape	*Lumbricidae*	CC *	✓	[23]
	*Chasmagnathus granulate*	CC *, LI, Ext	✓	[24,25,26]
	*Caenorhabditis elegans*	CC *, LI, Ext	✓	[27,28]
Gene expression	*Taeniopygia guttata*	CC	✓	[29]
Neophobia	*Rattus norvegicus*	CC *	✕	[21]
Orienting response				
Attentional capture	*Homo sapiens*	CC, Ext	✓	[30,31]
Skin conductance	*Homo sapiens*	CC	✕	[32,33]
Light approach	*Rattus norvegicus*	CC *, LI	✕	[19]
Head orienting	*Rattus norvegicus*	Ext	✓	[34]
Startle	*Rattus norvegicus*	CC *	✕	[34,35]
	*Mus musculus*	CC *	✕	[36]
Suppression				
Bar press	*Rattus norvegicus*	CC *	✓/✕	[20,34]
Licking	*Rattus norvegicus*	CC *, Ext	✓/✕	[34,37,38]
Wheel running	*Gallus gallus*	CC *	✓	[39,40]
Vasomotor activity	*Oryctolagus cuniculus*	Ext	✓	[8]

CC = Context change; LI = Latent inhibition; Ext = Extinction. * indicates that, to attenuate neophobia, animals were exposed to the new context before testing, as suggested in [19,20]. ✓ evidence; ✕ absence of evidence; ✓/✕ mixed results.
